# An Unusual Appearance of Double Right Coronary Artery

**DOI:** 10.4061/2010/123846

**Published:** 2010-09-29

**Authors:** Ahmet Akcay, Sedat Koroglu, Hakan Kaya, Murat Koleoglu, Gurkan Acar

**Affiliations:** Cardiology Department, Faculty of Medicine, Kahramanmaras Sutcu Imam University, 46050 Kahramanmaras, Turkey

## Abstract

Double right coronary artery (RCA) is an extremely rare coronary artery anomaly. We aimed to report an atherosclerotic double RCA which appeared after primary percutaneous intervention performed to treat acute inferior myocardial infarction. This is the first case in the literature, which the coronary arteries that can be accepted as double RCA have been hidden by total atherosclerotic occlusion of the proximal part of the RCA. In this paper, also the definition, correct diagnosis, and appropriate diagnostic methods for double RCA were discussed.

## 1. Introduction

Double right coronary artery (RCA) is a very rare coronary anomaly. It might be complicated with atherosclerosis and present with symptomatology of atherosclerosis. In this issue, we reported an atypical double RCA which appeared after primary percutaneous coronary intervention (PCI), performed to treat acute inferior myocardial infarction.

## 2. Case Report

**Figure 1:**
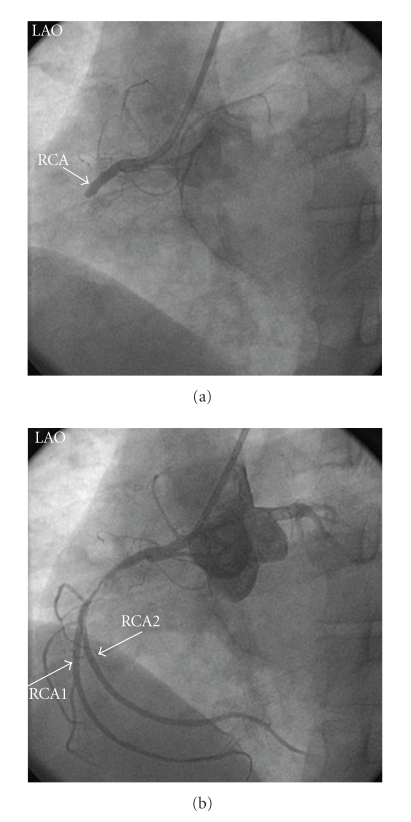
(a) LAO view of the totally occluded proximal RCA before percutaneous coronary intervention. (b) After predilatation with sprinter balloon, atypical double RCA was appeared (LAO: Left anterior oblique, RCA: Right coronary artery).

**Figure 2:**
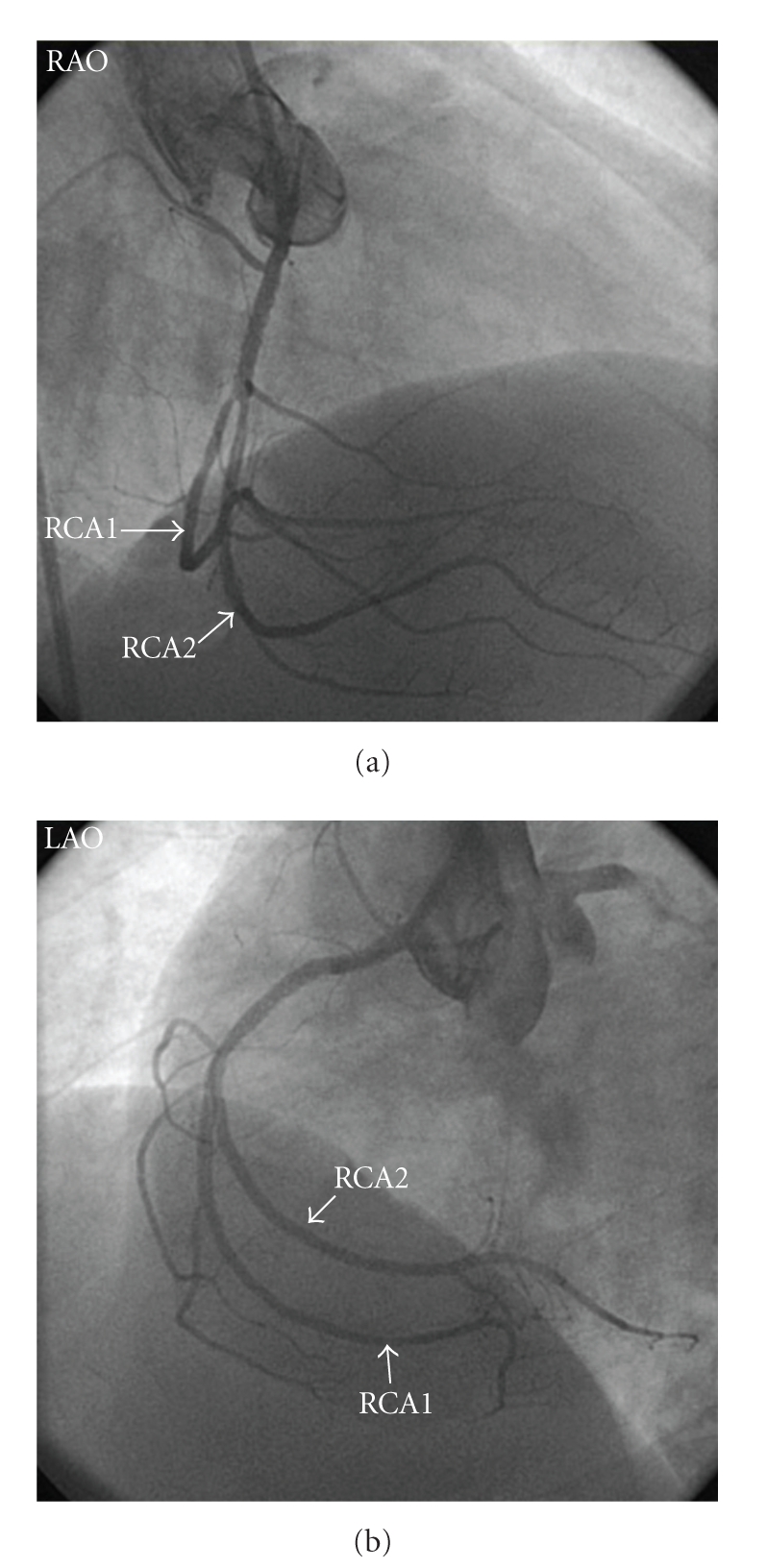
(a) RAO view, after implantation of bare metal stent. (b) LAO view, after implantation of bare metal stent (RAO: Right anterior oblique, LAO: Left anterior oblique).

40-year-old male was admitted to emergency department with retrosternal chest pain at rest for 1 hour. Physical examination was normal except for mild systolic hypertension. Electrocardiogram showed ST segment elevations in leads II, III, and aVF and reciprocal ST segment depressions in leads I and aVL. Coronary angiography showed total occlusion of the proximal RCA (Figure 1). The left coronary arteries were of normal origin and distribution; there were noncritical lesions in left anterior descending artery (LAD) and circumflex artery. Primary PCI was performed to the culprit RCA lesion by using bare metal stent. Control coronary angiogram demonstrated surprisingly that there were two parallel coronary arteries distal to the stent which could be diagnosed as atypical double RCA (Figures 1 and 2). He was administered tirofiban infusion, followed up for four day at intensive care unit and discharged without any complication.

## 3. Discussion

Double RCA is a very rare coronary anomaly which has been reported 21 times and in 27 cases in the literature [1–3]. The true definition and correct diagnosis of this uncommon anomaly remain controversial. Some authors have claimed that it is very difficult to distinguish double RCA with single orifice, from RCA which has a high take-that off of a large right ventricular artery, solely by coronary angiography. Nevertheless, they have mentioned that right anterior oblique (RAO) view provides better demonstration of artery courses and may be helpful in differentiating double RCA from a large right ventricular branch [4]. In their study, Sato et al. have proposed that double RCAs are defined when they supply the blood to the inferior left myocardium, thus both of the RCAs should course downwardly to reach the interventricular sulcus whether or not they cross the crux [5]. Eventually, double RCA was tried to be classified by an author from Turkey, where the most of the paper about this anomaly have been reported [6]. By using this reasonable classification we diagnosed our case as atypical double RCA.

In recent papers multidetector row computed tomography (MDCT) was offered as an alternative or adjunctive imaging method. MDCT allows 3D comprehension of the coronary artery system, and it is extremely useful to identify congenital coronary anomalies such as anomalous origin of the RCA. MDCT might also be useful to differentiate double RCA from high take off of a large branch [7]. 

Since the first report, this rare anomaly has been accepted as a benign entity. However, especially new reports in the last few years pointed out that double RCA might be complicated with atherosclerosis. Among 27 cases defined in the literature, atherosclerosis was determined in eight patients. Seven of them were presented with acute coronary syndromes, and three patients experienced inferior myocardial infarction. For the first case, coronary artery by-pass surgery was recommended, and elective PCI was performed for the second [8, 9]. The last case reported by Rohit et al. was a double RCA presenting with acute inferior wall infarction that managed with primary PCI. However, there has been no proved relationship; the increase in atherosclerotic double RCAs during the last few years was remarkable.

## 4. Conclusion

Definition and the diagnosis of double RCA remain controversial, but routine using of the RAO view and a promising imaging method, MDCT, may be helpful in such cases. Although this anomaly is generally considered as a benign entity, it may be atherosclerotic and can cause acute coronary syndromes including especially inferior myocardial infarction and may be associated with other anomalies. Invasive cardiologists should be kept in mind this possibility, because existence of such anomaly may change management strategies.

The appearance of this case is unique, because the coronary arteries that might be accepted as double RCA, probably atypical, have been hidden by total atherosclerotic occlusion of the proximal part of the RCA.
